# Clinical Outcomes of MRI-Guided Adaptive Brachytherapy for Each Fraction in Locally Advanced Cervical Cancer: A Single Institution Experience

**DOI:** 10.3389/fonc.2022.841980

**Published:** 2022-03-17

**Authors:** Yunbo Chi, Ying Pan, Ning Zhang, Dongmei Han, Xin Guo, Zhuang Mao, Guanghui Cheng

**Affiliations:** ^1^Department of Radiation Oncology, China-Japan Union Hospital of Jilin University, Changchun, China; ^2^Department of Gynecology, China-Japan Union Hospital of Jilin University, Changchun, China

**Keywords:** locally advanced cervical cancer, magnetic resonance imaging guided adaptive brachytherapy, intracavitary brachytherapy, interstitial brachytherapy, hybrid intracavitary/interstitial brachytherapy, clinical outcome

## Abstract

**Purpose:**

This study aims to evaluate clinical outcomes of MRI-guided adaptive brachytherapy (MR-IGABT) for each brachytherapy fraction in patients with locally advanced cervical cancer (LACC).

**Methods and Materials:**

A retrospective analysis was performed on 97 consecutive patients with LACC treated with 44.0–50.4 Gy external beam radiotherapy (EBRT) ± concurrent platinum-containing chemotherapy followed by 4 × 7 Gy MR-IGABT between September 2014 and April 2019. Intracavitary (IC)/interstitial (IS)/hybrid intracavitary and interstitial (IC/IS) brachytherapy was used in MR-IGABT. Brachytherapy planning and dose reporting followed the GEC-ESTRO recommendations. Clinical outcomes including overall survival (OS), cancer-specific survival (CSS), progression-free survival (PFS), local control (LC), and treatment-related toxicity evaluated by the RTOG criteria were analyzed. Kaplan–Meier and univariable and multivariable Cox regression analyses were used to analyze the prognostic factor.

**Results:**

Median follow-up was 21.1 months. Median dose to 90% (D_90_) of the high-risk clinical target volume (HR-CTV) was 91.7 Gy (range 76.7~107.2 Gy). Two-year OS, CSS, PFS, and LC were 83.5%, 84.1%, 71.1%, and 94.8%, respectively. Four patients (4.1%) suffered from grade 3 late gastrointestinal radiation toxicity, and no other grade 3 or greater radiation toxicity occurred. Initial HR-CTV was an independent factor of OS (*p* = 0.001, HR = 1.018/cm^3^), PFS (*p* = 0.012, HR = 1.012/cm^3^), and LC (*p* = 0.011, HR = 1.028/cm^3^). The HR-CTV D_90_ (*p* = 0.044, HR = 0.923/Gy) was an independent factor of PFS. Age was an independent factor of LC (*p* = 0.010, HR = 1.111/year).

**Conclusion:**

For patients with LACC, MR-IGABT was effective and safe. It showed favorable LC, OS, and minimal toxicity. Moreover, initial HR-CTV, HR-CTV D_90_, and age were significant prognostic factors.

## Introduction

In global cancer statistics, cervical cancer ranks fourth for both incidence and mortality in women (1). In China, cervical cancer had a significant upward trend in age-standardized incidence rates (2).

Stages IB2, IIA2, IIB, IIIA, IIIB, and IVA (FIGO 2009) cervical cancers are all locally advanced cervical cancer (LACC). To treat this type of cervical cancer, the National Comprehensive Cancer Network (NCCN) guidelines recommend the external beam radiotherapy (EBRT), concurrent platinum-containing chemotherapy, and brachytherapy (category 1) (3). As a critical component of the definitive radiation therapy, brachytherapy technology has been rapidly developing in recent years. In consideration of significant changes in the tumor regresses and the topography of the target and organs at risk during the course of treatment (4), image-guided adaptive brachytherapy (IGABT) became an individualized treatment method for patients with LACC. IGABT improves overall survival (OS) and generates a high rate of local tumor control (LC) with a moderate rate of treatment-related morbidity (5–8). IGABT has been developing particularly in Europe, North America, and Asia (9).

The preferred imaging technologies for IGABT for LACC are CT and MRI. Compared with CT, advantages of MRI lie in the soft tissue contrast and in discrimination of cervical cancer from normal uterine and adjacent tissue (10). This helps to define the tumor shrinkage and topography after EBRT (11). The NCCN guidelines recommend MRI as the best imaging modality to determine soft tissue and parametrial involvement in patients with advanced tumors (3). Even so, it is difficult for every institution to gain MRI access for each individual brachytherapy fraction (7, 12–17). Due to the limited MRI availability, some institutions use MRI only in some of the brachytherapy fractions (12, 16–18). The use of MRI-guided adaptive brachytherapy (MR-IGABT) in each fraction is still limited (18). The aim of this study was to evaluate the clinical outcomes of MR-IGABT in each fraction for Chinese patients with LACC.

## Materials and Methods

### Patients

Ninety-seven consecutive patients were included in this retrospective study, treated between September 2014 and April 2019. The following eligibility criteria were applied: patients with stages IB2 to IVA (FIGO 2009), who underwent the MR-IGABT (4 × 7 Gy) in our institution and did not have a previous history of malignancy. The present study was approved by the ethics committee of our institution.

### Treatment

All patients received EBRT to the pelvis with and without concurrent platinum-containing chemotherapy as described below, followed by 4 × 7 Gy MR-IGABT. Each brachytherapy fraction was guided by T2-weighted (T2W) MRI.

Seventy-five (77.3%) patients underwent concurrent platinum-containing chemotherapy, 49 (50.5%) patients were administered platinum drugs as a single agent, and 26 (26.8%) patients were administered platinum combined with paclitaxel or docetaxel (**Table 1** shows the patient information). EBRT (Synergy; Elekta AB, Stockholm, Sweden) used three-dimensional conformal radiotherapy (3D-CRT) or intensity modulated radiotherapy (IMRT), with a total prescribed dose of 44.0~50.4 Gy in 1.8~2.0 Gy fractions, with some patients receiving a pelvic nodal boost.

**Table 1:** Patients and treatment characteristics.

Characteristic	
Total number of patients	97
Median age, years (range)	54 (30~79)
FIGO stage [*n* (%)]	
IB2	3 (3.1)
IIA2	13 (13.4)
IIB	58 (59.8)
IIIA	4 (4.1)
IIIB	15 (15.5)
IVA	4 (4.1)
Histology [*n* (%)]	
Squamous cell carcinoma	92 (94.9)
Adenocarcinoma	4 (4.1)
Clear cell carcinoma	1 (1.0)
Lymph node status	
Positive	25 (25.8)
Negative	72 (74.2)
EBRT dose/fraction [*n* (%)]	
44 Gy/22f	1 (1.0)
45 Gy/25f	84 (86.6)
46 Gy/23f	3 (3.1)
50 Gy/25f	5 (5.2)
50.4 Gy/28f	4 (4.1)
EBRT technique [*n* (%)]	
3D-CRT	34 (35.1)
IMRT	63 (64.9)
Concurrent chemotherapy [*n* (%)]	
Yes	75 (77.3)
No	22 (22.7)
Brachytherapy technique [*n* (%)]	
Solely IC brachytherapy	7 (7.2)
IC/IS or IS brachytherapy	90 (92.8)
Median overall treatment time [days (range)]	63 (40~141)

**Figure 1:**
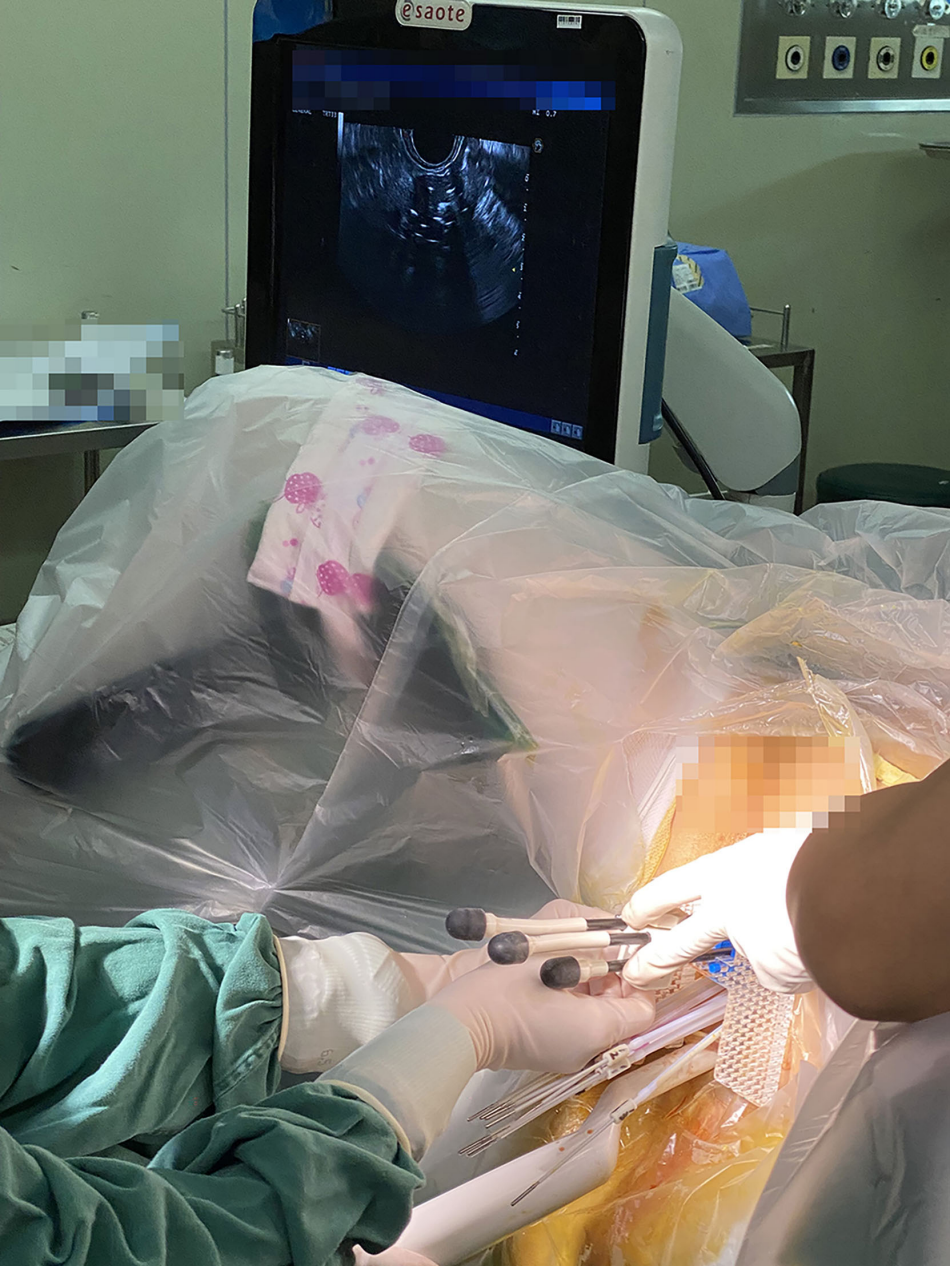
Use of hybrid intracavitary and interstitial (IC/IS) brachytherapy with assistant of real-time transrectal ultrasound. All the implants were MR compatible.

**Figure 2:**
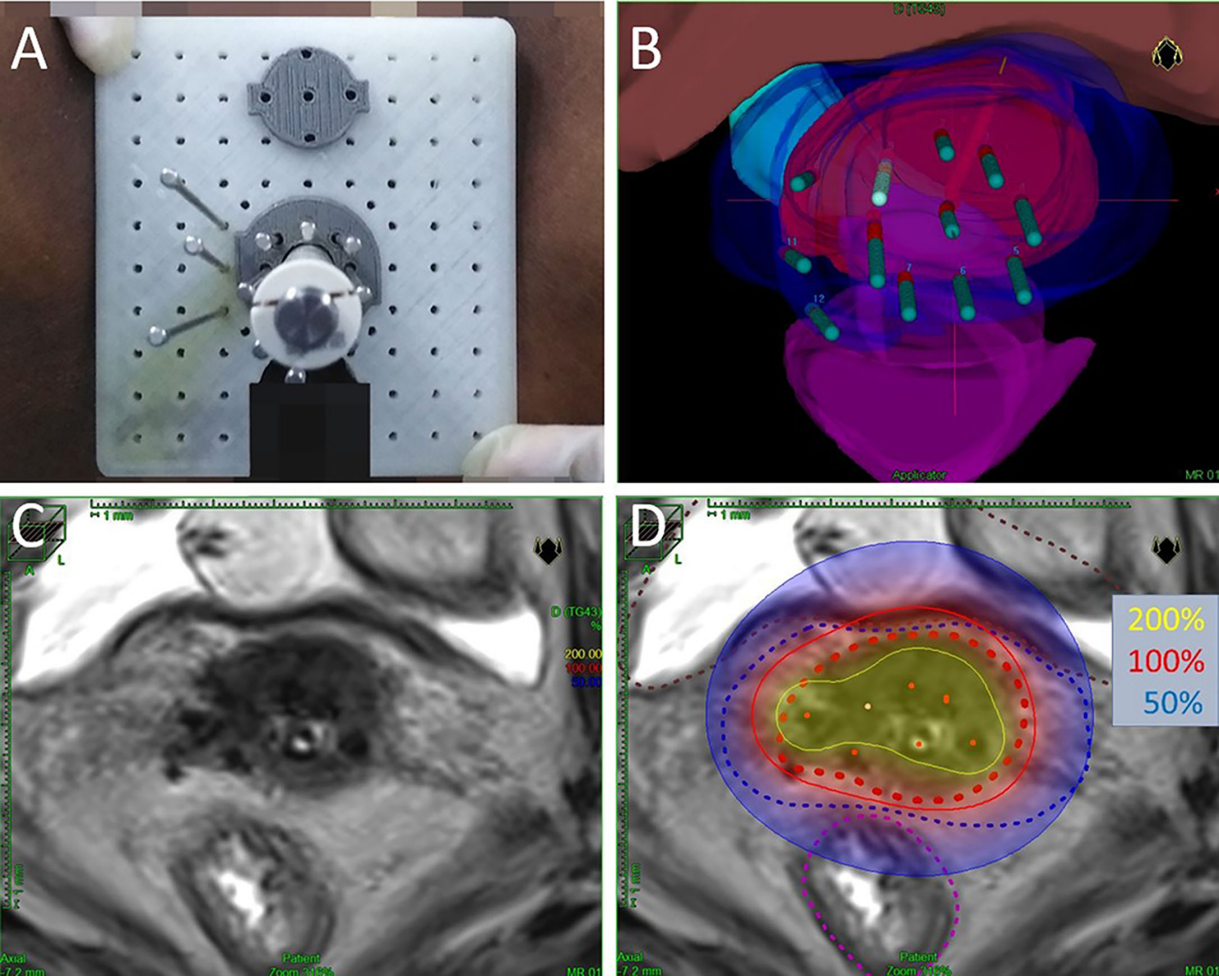
**(A)** Macroscopic view of self-made 3D-Printed applicator. **(B)** Three-dimensional view of the same implant planning data. The volumes represent HR-CTV (red), IR-CTV (blue), bladder (pink), rectum (purple), and sigmoid (cyan). **(C)** Axial view of T2-weighted magnetic resonance images (with implant *in situ*). **(D)** Axial view of brachytherapy dose distribution. Dotted red line is HR-CTV, dotted blue line is IR-CTV, dotted brown line represents the bladder, and dotted purple line represents the rectum. The isodose lines color code conventions are: solid yellow line = 200%; solid red line = 100%; solid blue line = 50% per treatment fraction.

Each brachytherapy fraction utilizes ultrasound-assisted applicator/catheter insertion under general anesthesia (**Figure 1**). Applicator included Utrecht interstitial Fletcher CT/MRI Applicator Set (Elekta AB, Stockholm, Sweden), Interstitial Ring CT/MRI Applicator Set (Elekta AB, Stockholm, Sweden), Vaginal CT/MRI Multi Channel Applicator Set (Elekta AB, Stockholm, Sweden), self-made 3D-Printed applicator (This type of 3D-printed applicator consists of tandem, vaginal cylinder, and perineum template). Utrecht applicator and Ring applicator were appropriate for bulky disease. Multi Channel Applicator was appropriate for patients with vagina involvement. However, due to the invariable positions of the channel on the vaginal templates of the Utrecht applicator or Ring applicator, patients with bulky infiltrative extensive disease or narrow vagina cannot achieve the prescription dose. Self-made 3D-printing template and freehand interstitial technique provide the right and flexible position choices to make an adequate dose coverage (**Figure 2**).

A total of 388 brachytherapy fractions were included. Sixty-seven (17%) fractions used intracavitary (IC) brachytherapy. Three hundred and 21 (83%) fractions used hybrid intracavitary and interstitial (IC/IS) brachytherapy or interstitial (IS) brachytherapy alone. The IS brachytherapy alone was performed in a few brachytherapy fractions (6/388) with the obstruction of cervical canal orifice which were not appropriate to use tandem. In addition, 80 (20.6%) fractions used freehand interstitial brachytherapy, and for 90 patients (92.8%), the IC/IS or IS technique was used.

3.0-T MRI scans (Siemens Skyra, Erlangen, Germany) were performed after recovery from anesthesia (with implant *in situ*). T2W MRI of each brachytherapy fraction was used for the delineation of target volume and organs at risk (OARs), as referred to in the GEC-ESTRO recommendations (19, 20). High-dose rate (HDR) iridium-192 after-loading therapy (Microselectron V2 HDR; Nucletron, Veenendaal, The Netherlands. Treatment Planning System Oncentra V4.3; Nucletron, Veenendaal, The Netherlands) was applied to each brachytherapy fraction.

The equivalent dose based on linear-quadratic model in 2 Gy fraction (EQD_2_), with α/β of 10 Gy for tumor and 3 Gy for OARs, was used to calculate the cumulative doses from EBRT and MR-IGABT. Dosimetric parameters were evaluated after the GEC-ESTRO recommendations (19, 20).

### Follow-Up and Endpoints

All patients were followed up by periodical check-up which consists of bimanual pelvic examination and imaging studies (pelvic MRI or CT scan) every 3 months in the first 2 years, at 6 months intervals for the next 3 years, and then annually.

The RECIST guidelines (version 1.1) (21) were used to evaluate the initial tumor response. Overall survival (OS) and cancer-specific survival (CSS) were defined as the period from the date of diagnosis until the date of death and death by cervical cancer, respectively. Progression-free survival (PFS) was defined as the period from diagnosis to the date of first documented evidence of progressive or recurrent disease or death. Local control (LC) was defined as the period from the diagnosis to the date of local relapse. Acute radiation morbidity and late radiation morbidity were evaluated by the RTOG morbidity criteria (22). Severe toxicity was defined as grades 3–5 complications.

### Statistical Analysis

Statistical analysis was performed using SPSS (version 24). Continuous variables and classification variables were described as medians (ranges) and counts (percentages), respectively. Continuous variables were compared using Student’s *t*-test or rank-sum test. The correlations were analyzed using Pearson’s or Spearman’s correlation. The survival curves were performed using the Kaplan–Meier method. Univariable factors were evaluated using log-rank tests and Cox regression analysis. Multivariable factors were evaluated with Cox regression analysis. *p* < 0.05 was considered statistically significant.

## Results

A total of 97 consecutive patients were included in this study, treated between September 2014 and April 2019. The median age at diagnosis was 54 (range 30~79) years. The median overall treatment time (OTT) was 63 days (range 40~141 days). **Table 1** shows patients and treatment characteristics. For stages IIA2~IVA tumors, IC/IS and IS brachytherapy techniques were used in a higher proportion of 65%, 84%, 81%, 100%, and 94%, respectively.

### Dose–Volume Parameters

Dosimetric outcomes are presented in **Table 2**. The median HR-CTV D_90_, HR-CTV D_98_, HR-CTV D_100_, intermediate-risk CTV (IR-CTV) D_90_, and IR-CTV D_100_ were 91.7 Gy (76.7~107.2 Gy), 81.7 Gy (69.2~92.5 Gy), 71.2 Gy (63.0~82.1 Gy), 67.0 Gy (60.4~75.0 Gy), and 56.6 Gy (51.6~62.3 Gy), respectively. The initial, second, third, and fourth HR-CTV (range) were 32.6 cm^3^ (9.3~221.0 cm^3^), 31.0 cm^3^ (10.9~115.8 cm^3^), 28.5 cm^3^ (9.9~103.3 cm^3^), and 29.6 cm^3^ (8.9~118.8 cm^3^), respectively.

**Table 2:** Dosimetric outcomes.

Parameters	Median	Range
HR-CTV D_90_ (Gy)	91.7	76.7~107.2
HR-CTV D_98_ (Gy)	81.7	69.2~92.5
HR-CTV D_100_ (Gy)	71.2	63.0~82.1
IR-CTV D_90_ (Gy)	67.0	60.4~75.0
IR-CTV D_100_ (Gy)	56.6	51.6~62.3
Initial HR-CTV (cm^3^)	32.6	9.3~221.0
The second HR-CTV (cm^3^)	31.0	10.9~115.8
The third HR-CTV (cm^3^)	28.5	9.9~103.3
The fourth HR-CTV (cm^3^)	29.6	8.9~118.8
Bladder		
D_0.1cc_ (Gy)	94.2	69.0~116.5
D_1cc_ (Gy)	83.0	63.3~95.0
D_2cc_ (Gy)	77.4	60.7~89.6
Rectum		
D_0.1cc_ (Gy)	80.0	59.7~98.8
D_1cc_ (Gy)	69.3	52.7~86.1
D_2cc_ (Gy)	65.3	50.9~80.3
Sigmoid		
D_0.1cc_ (Gy)	79.7	56.8~105.0
D_1cc_ (Gy)	70.0	52.6~78.0
D_2cc_ (Gy)	65.6	51.0~71.8
Small bowel		
D_0.1cc_ (Gy)	76.5	47.3~101.6
D_1cc_ (Gy)	66.9	46.1~81.5
D_2cc_ (Gy)	63.3	45.7~75.1

### Treatment Outcomes

**Figure 3:**
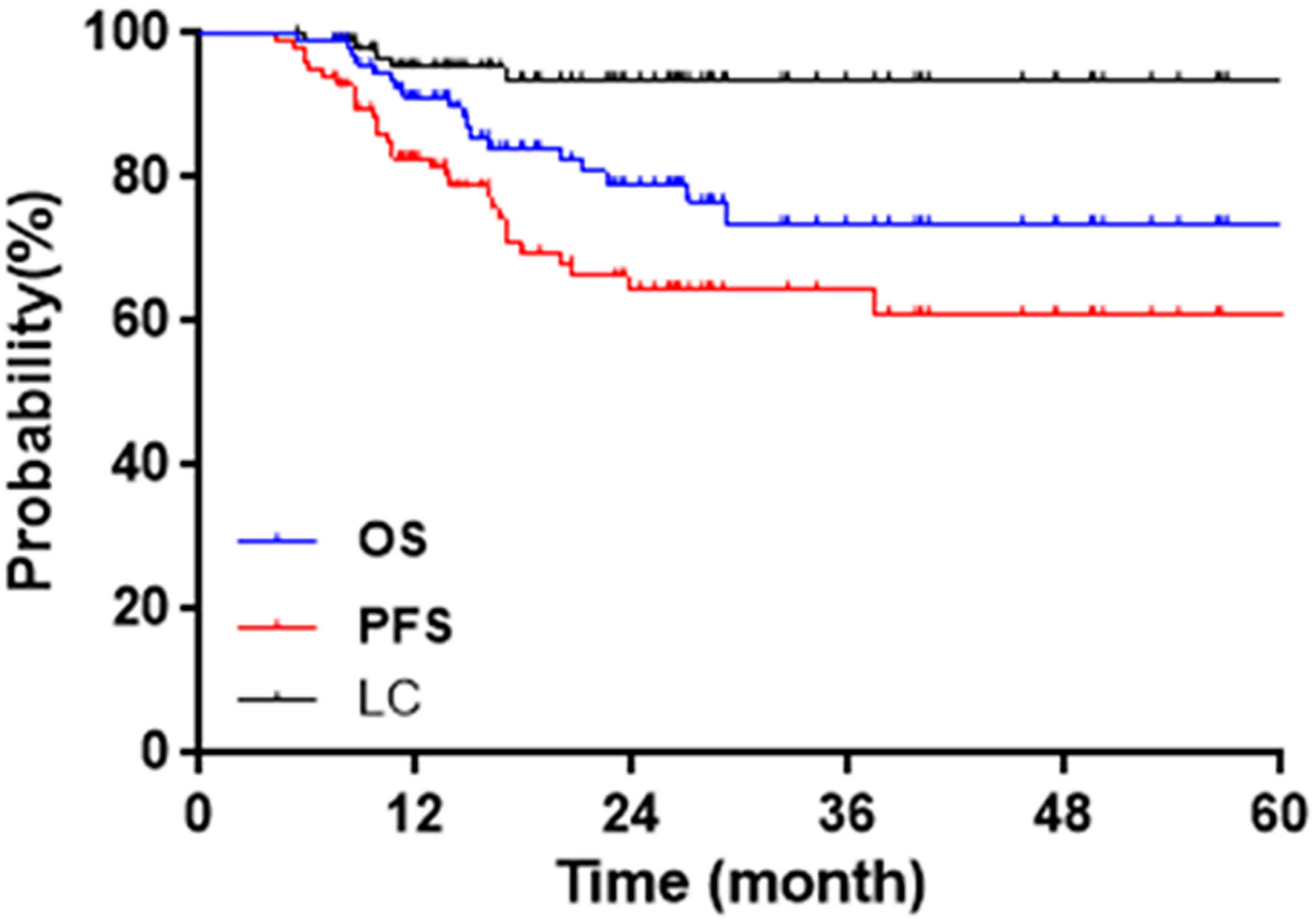
Kaplan–Meier curves for OS, PFS, and LC.

The median follow-up was 21.1 months (5.4~67.0 months). The initial tumor responses were 64 complete responses (CR) and 33 partial responses (PR). CR + PR were achieved in 97/97 (100%) patients. Two-year OS, CSS, PFS, and LC were 83.5%, 84.5%, 71.1%, and 94.8%, respectively. **Figure 3** shows Kaplan–Meier curves for OS, PFS, and LC.

Eighteen patients have died, 17 from cervical cancer, 1 from a nontumor cause. The leading cause of the nontumor-caused death (OS = 16.0 months) was infection. This patient’s last physical examination showed positive hemoculture, without tumor recurrence, or digestive tract fistula. Therefore the death of this patient was not caused by cervical cancer or radiotherapy-related toxicity. Five patients suffered from local failures. Two-year LC was 100% for IB2, 92.3% for IIA2, 98.3% for IIB, 75% for IIIA, 93.3% for IIIB, and 75% for IVA. A total of 29 events occurred in PFS: 5 local failures (1 with pelvic metastasis), 2 pelvic metastasis, 20 distant metastasis (4 with pelvic metastasis), and 2 deaths.

Outcomes of univariable and multivariable analyses are shown in **Tables 3**–**5**. Univariable analyses show: HR-CTV D_90_, HR-CTV D_98_, each fraction of HR-CTV, and initial HR-CTV >40 cm^3^ showed a statistical difference in OS. HR-CTV D_90_, HR-CTV D_90_ ≥87 Gy, HR-CTV D_98_, each fraction of HR-CTV, and initial HR-CTV >40 cm^3^ showed a statistical difference in PFS. HR-CTV D_90_ ≥87 Gy, each fraction of HR-CTV, and initial HR-CTV >40 cm^3^, the initial tumor response showed a statistical difference in LC.

**Table 3:** Univariable analyses (classification variables).

	OS				PFS				LC			
	Event	Censored data	%	*p*	Event	Censored data	%	*p*	Event	Censored data	%	*p*
Stage				0.082				0.178				0.056
IB2	0	3	100%		0	3	100%		0	3	100%	
IIA2	3	10	76.9%		4	9	69.2%		1	12	92.3%	
IIB	9	49	84.5%		15	43	74.1%		1	57	98.3%	
IIIA	1	3	75%		2	2	50%		1	3	75%	
IIIB	3	12	80%		6	9	60%		1	14	93.3%	
IVA	2	2	50%		2	2	50%		1	3	75%	
Histology				0.781				0.409				0.876
Squamous cell carcinoma	17	75	81.5%		27	65	70.7%		5	87	94.6%	
Adenocarcinoma	1	3	75%		2	2	50%		0	4	100%	
Others	0	1	100%		0	1	100%		0	1	100%	
Lymph node status				0.110				0.058				0.931
Positive	6	19	76%		9	16	64%		1	24	96%	
Negative	12	60	83.3%		20	52	72.2%		4	68	94.4%	
Concurrent chemotherapy				0.378				0.967				0.359
Yes	12	63	84%		22	53	70.7%		3	72	96.0%	
No	6	16	72.7%		7	15	68.2%		2	20	90.9%	
HR-CTV D_90_				0.106				0.015				0.039
≥87 Gy	14	71	83.5%		22	63	74.1%		3	82	96.5%	
<87 Gy	4	8	66.7%		7	5	41.7%		2	10	83.3%	
Initial HR-CTV (cm^3^)				<0.001				<0.001				0.001
>40 (cm^3^)	13	20	60.6%		18	15	45.5%		5	28	84.8%	
≤40 (cm^3^)	5	59	92.2%		11	53	82.8%		0	64	100%	
OTT				0.910				0.807				0.162
>8 weeks	13	56	81.2%		21	48	69.6%		5	64	92.8%	
≤8 weeks	5	23	82.1%		8	20	71.4%		0	28	100%	
Initial tumor response				0.728				0.809				0.040
CR	10	54	84.4%		18	46	71.9%		1	63	98.4%	
PR	8	25	75.8%		11	22	66.7%		4	29	87.9%	

**Table 4:** Univariable analyses (continuous variables).

	OS		PFS		LC	
	HR (95% CI)	*p*	HR (95% CI)	*p*	HR (95% CI)	*p*
Age (per year)	–	0.855	–	0.724	–	0.077
HR-CTV D_90_ (per Gy)	0.873 (0.758~0.956)	0.003	0.892 (0.833~0.956)	0.001	–	0.093
HR-CTV D_98_ (per Gy)	0.891 (0.805~0.986)	0.026	0.910 (0.841~0.984)	0.018	–	0.406
HR-CTVD_100_ (per Gy)	–	0.085	–	0.073	–	0.570
IR-CTV D_90_ (per Gy)	–	0.253	–	0.413	–	0.056
IR-CTV D_100_ (per Gy)	–	0.922	–	0.538	–	0.067
Initial HR-CTV (per cm^3^)	1.019 (1.011~1.028)	<0.001	1.015 (1.007~1.022)	<0.001	1.022 (1.006~1.038)	0.006
The second HR-CTV (per cm^3^)	1.031 (1.016~1.046)	<0.001	1.025 (1.013~1.037)	<0.001	1.035 (1.009~1.061)	0.009
The third HR-CTV (per cm^3^)	1.042 (1.024~1.061)	<0.001	1.033 (1.018~1.048)	<0.001	1.048 (1.017~1.081)	0.003
The fourth HR-CTV (per cm^3^)	1.028 (1.013~1.042)	<0.001	1.019 (1.007~1.032)	0.002	1.030 (1.005~1.056)	0.019
OTT (per day)	–	0.374	–	0.947	–	0.098

**Table 5:** Multivariable analyses.

	OS				PFS				LC			
	*p*	*B*	Wald x^2^	HR (95% CI)	*p*	*B*	Wald x^2^	HR (95% CI)	*p*	*B*	Wald x^2^	HR (95% CI)
Age (years)	NS	–	–	–	NS	–	–	–	0.010	0.106	6.616	1.111 (1.025~1.205)
HR-CTV D_90_ (per Gy)	NS	–	–	–	0.044	−0.080	4.052	0.923 (0.853~0.998)	NS	–	–	–
Initial HR-CTV (per cm^3^)	0.001	0.018	10.102	1.018 (1.007~1.029)	0.012	0.012	6.249	1.012 (1.003~1.021)	0.011	0.028	6.451	1.028 (1.006~1.051)

NS = p > 0.05.

Multivariable analyses show the initial HR-CTV was an independent factor of OS (*p* = 0.001, HR = 1.018/cm^3^, 95% CI = 1.007~1.029), PFS (*p* = 0.012, HR = 1.012/cm^3^, 95% CI = 1.003~1.021), and LC (*p* = 0.011, HR = 1.028/cm^3^, 95% CI = 1.006~1.051). The HR-CTV D_90_ (*p* = 0.044, HR = 0.923/Gy, 95% CI = 0.853~0.998) was an independent factor of PFS. Age was an independent factor of LC (*p* = 0.010, HR = 1.111/year, 95% CI=1.025~1.205).

### Toxicity

Four patients (4.1%) suffered from grade 3 late gastrointestinal radiation toxicity, and no other severe acute or late radiation toxicity occurred. **Table 6** shows the distribution of different types of radiation toxicity. In addition, during the operation using interstitial technology, no serious bleeding or infections occurred.

**Table 6:** The distribution of different types of radiation morbidity (*n*, %).

Type of radiation morbidity	Grade 0	Grade 1	Grade 2	Grade 3
Acute mucous membrane	33 (34.0)	57 (58.8)	7 (7.2)	0
Acute bladder	79 (81.4)	16 (16.5)	2 (2.1)	0
Acute lower gastrointestinal	90 (92.8)	5 (5.2)	2 (2.1)	0
Late bladder	78 (80.4)	16 (16.5)	3 (3.1)	0
Late gastrointestinal	79 (81.4)	10 (10.3)	4 (4.1)	4 (4.1)

## Discussion

MRI has been recommended as gold standard imaging for cervical cancer contours, with some comparative studies previously published (14, 23, 24), and for MR-IGABT (repetitive MRI during complete brachytherapy treatment), several studies reported its clinical efficacy for LACC patients in Europe (7, 25) and North America (26). This study aimed to report the treatment outcomes of MR-IGABT for 97 Chinese LACC patients.

Whether MR-IGABT brings satisfactory clinical outcomes for cervical cancer, it has been a research priority of many radiotherapy centers in recent years. Lindegaard et al. (6) compared outcomes of LACC between 2D (X-ray)-guided brachytherapy and MR-IGABT. The 3-year OS of MR-IGABT showed a significant improvement (79% vs. 63%, *p* = 0.005), and 3-year LC of MR-IGABT was achieved in 91% of patients. Moreover, the moderate and severe late morbidity were both reduced by about 50% (*p* = 0.02). Kamran et al. (27) compared outcomes of LACC of MR-IGABT versus CT-guided brachytherapy. OS was significantly improved in MR-IGABT (84% vs. 56%, *p* = 0.036), and 2-year LC were 96% and 87% (*p* = 0.65), respectively. A large multicenter cohort study of Retro EMBRACE (28) included 731 LACC patients showed the efficacy and safety of MR-IGABT. Five hundred and ninety-two (80.9%) patients used MR-IGABT for at least one brachytherapy fraction, and 168 (23.0%) patients used IC/IS technique. The 3/5-year actuarial OS and LC were 74%/65% and 91%/89%, respectively. The 3/5-year grades 3–5 late morbidity was 4%/5% and 6%/7% for bladder and gastrointestinal tract, respectively. These excellent outcomes of MR-IGABT have been demonstrated in the western world. For Chinese patients with LACC, Wu et al. (18) recently evaluated the clinical outcomes of MR-IGABT where MRI was repeated at each implant (with implant *in situ*), in limited patient numbers (49 Chinese patients), with the first and the third brachytherapy fractions using MR-IGABT and other brachytherapy fractions were planned on CT imaging. Two-year OS and LC were both achieved in 90% of the patients with no severe late toxicity.

**Table 7** (5–8, 17, 18, 25–30) summarizes the clinical outcomes of IGABT mentioned in this study and/or other studies recently published. The clinical outcomes of MR-IGABT, including the present study, show favorable OS, LC, and limited severe morbidity. The 2-year rates for OS and LC were achieved in 83.5% and 94.8% in the present study. Studies (8, 31) showed most local failures occurring less than 2 years after treatment. Tan et al. (32) summarized the distribution of local failure by time: 44.9% (year 1), 29.0% (year 2), 8.7% (year 3), 8.7% (year 4), 2.9% (year 5), 1.4% (years 6–10), and 4.3% (>10 years).

**Table 7:** Clinical outcomes of IGABT reported in studies.

Study	IGABT technique	No. of patients	OS	LC
Pötter et al. (7)	MRI	156	68% (3-year)	95% (3-year)
Charra-Brunaud et al. (8)	CT/MRI (group 3, 3D arm)	117	74% (2-year)	78.5% (2-year)
Lindegaard et al. (6)	MRI (group MR-IGABT)	140	79% (3-year)	91% (3-year)
Nomden et al. (25)	MRI	46	65% (3-year)	93% (3-year)
Rijkmans et al. (5)	CT/MRI^a^ (group IGABT)	83	86% (3-year)	–
Sturdza et al. (28)	CT/MRI^b^	731	65% (5-year)	89% (5-year)
Kamran et al. (27)	MRI (group MR-IGABT)	29	84% (2-year)	96% (2-year)
van Dyk et al. (17)	MRI/US^c^	191	63% (5-year)	86% (5-year)
Horeweg et al. (29)	CT/MRI^d^	155	65.9% (5-year)	90.4% (5-year)
Wu et al. (18)	CT/MRI	49	90% (2-year)	90% (2-year)
Horne et al. (26)	MRI	239	72.7% (5-year)	90.8% (5-year)
Gill et al. (30)	CT/MRI^e^	128	85% (2-year)	92% (2-year)
Present study	MRI	97	83.5% (2-year)	94.8% (2-year)

a In group IGABT, 48.2% of patients underwent MRI scanning for all fractions, 38.6% of patients underwent MRI and CT for different fractions, and 13.3% of patients underwent only CT. Pelvic recurrence was found in 7% at 3 years for the MR-IGABT group.

b In this study, 80.9% of patients underwent MR-IGABT for at least one fraction and for 19.1% of patients, only CT was used.

c All patients underwent MRI and transabdominal ultrasound imaging with applicators in situ at the first brachytherapy fraction and ultrasound imaging alone at subsequent fractions.

d In this study, 72.3% MRI scanning was used for all fractions, 23.9% of patients underwent MRI and CT for different fractions, and 3.9% of patients underwent only CT.

e All patients underwent MR-IGABT for at least one fraction.

PFS is another important clinical outcome. Two-year PFS in the present study was 71.1%. A total of 20 distant metastasis occurred, which was the largest share of PFS (20/29). This was similar to other studies (7, 32, 33). Potter et al. (7) reported that IGABT technology significantly reduces local failure, which will further make distant metastases the predominant failure pattern. In order to reduce the risk of distant metastases and improve PFS, intensified chemoradiotherapy (34) or other therapy (such as molecular targeted therapy or immunotherapy) should be taken into account.

The most common radiation toxicity that occurred was grade 1 acute mucous membrane radiation toxicity (58.1%). Other incidences of radiation toxicity (acute bladder, acute lower gastrointestinal, late bladder, and late gastrointestinal) were all less than 20% (19.4%, 9.2%, 17.3%, and 19.4%, respectively). Only 4 patients (4.1%) showed grade 3 late gastrointestinal radiation toxicity. After symptomatic treatment, 2 patients fell to grade 1 and 2 patients fell to grade 0. ABS guideline (35) recommended the D_2cc_ to the bladder, rectum, and sigmoid are ≤90, ≤75, and ≤75 Gy, respectively. In this study, only 2 (2%) patients had a rectum D_2cc_ higher than 75 Gy. The interstitial-related side effect (such as pain, bleeding, infection) was settled with symptomatic treatment. MR-IGABT with interstitial technique can fully conform to dose limits for OARs, which will further lead to a well- tolerated treatment.

In the present study, the larger initial HR-CTV (per cm^3^) was an independent factor for worse OS, PFS, and LC. We investigated the correlations between initial HR-CTV and other dose–volume parameters. The initial HR-CTV was negatively correlated with HR-CTV D_90_ (*p* = 0.002), HR-CTV D_98_ (*p* = 0.016), and HR-CTVD_100_ (*p* = 0.006) and positively correlated with bladder D_0.1cc_ (*p* = 0.047), bladder D_1cc_ (*p* < 0.001), bladder D_2cc_ (*p* < 0.001), rectum D_0.1cc_ (*p* = 0.027), rectum D_1cc_ (*p* < 0.001), rectum D_2cc_ (*p* < 0.001). The initial HR-CTV showed no correlation between the dose to sigmoid and small bowel. The probable cause is the geometrical uncertainties in OARs (36) (e.g., filling status and motion in relation to the radiation sources). These uncertainties are highly related to the motility of the organs, such as, the sigmoid and small bowel. The high dose of HR-CTV D_90_ was an independent prognostic factor for improved PFS. Furthermore, we found that age was an independent factor for LC. A large national cohort analysis (37), which included 24,126 patients found that age was an independent predictor for the receipt of complete treatment (concurrent chemotherapy with combination external beam radiation and brachytherapy to total dose ≥70 Gy) for cervical cancer. Its multivariable analysis showed age groups of women older than 61 (group 61–70, group 71–80, group 80+) were less likely to be treated with complete treatment. In the present study, age was significantly different (*p* = 0.016) between patients with and without concurrent chemotherapy [median age was 53 (range 30–76) vs. 61.5 (range 33–79)]. The older age resulted in incomplete treatment, which may influence the LC.

The independent prognostic factors for LC in previous studies (26, 38, 39) where patients received MR-IGABT have reported stage, histology, HR-CTV D_90_, and initial HR-CTV, OTT. Dimopoulos et al. (39) found that LC was clearly greater if the HR-CTV D_90_ ≥87 Gy. Horne et al. (26) found that LC was affected by HR-CTV >40 cm^3^. In the present study, 2-year LC was 96.5% for HR-CTV D_90_ ≥87 Gy versus 83.3% for HR-CTV D_90_ <87 Gy (log-rank, *p* = 0.039) and 100% for initial HR-CTV ≤40 cm^3^ versus 84.8% for initial HR-CTV >40 cm^3^ (log-rank, *p* = 0.001). In addition, we found that 2-year LC was significantly different between CR and PR (98.4% for CR vs. 87.9% for PR, log-rank, *p* = 0.040), indicating the initial tumor response may influence LC.

Interstitial techniques including IC/IS and IS techniques were used as a dose-escalation method in 90 of 97 patients in this study, with 85 patients (87.6%) HR-CTV D_90_ ≥87 Gy and 96 patients (99.0%) HR-CTV D_90_ ≥80 Gy. The median HR-CTV D_90_ was 91.7 Gy, which meet the ABS (35) and NCCN guidelines (3). Only 1 patient (1%) showed HR-CTV D_90_ lower than 80 Gy, due to the large volume of initial HR-CTV (93.08 cm^3^). Compared with traditional IC brachytherapy alone, interstitial technique is more feasible with adequate coverage of disease in the vagina and parametrium (40).

In the present study, 69 patients (71.1%) had OTT longer than 8 weeks, which was suggested within 8 weeks (3). The main reason was patients did not receive timely brachytherapy treatment after the end of EBRT, which can be further optimized. The reduction of OTT to avoid repopulation in cervical cancer is known to be one of the ways to improve LC. Compared with the difficulty to deliver a higher dose of radiation to cervical cancer, OTT can be more easily kept within certain limits. Mazeron et al. (41) reported that the inverse correlation (probit model) between overall treatment time and local control and excessive OTT was an independent factor of LC for LACC treated by IGABT, with a cutoff of 55 days (Log-rank, *p* = 0.004, and Cox model, *p* = 0.047). Although, for OTT, there was no statistical significance in OS, PFS, and LC in the present study, we found OTT was significantly different (*p* = 0.035) between CR and PR [median OTT was 62 days (range 40–125 days) vs. 64 days (range 54–141 days)]. In consideration of the significant difference of 2-year LC between CR and PR in this study, the longer OTT results in a poorer initial tumor response, which further impacts LC. However, there is still insufficient high-level evidence with regard to OTT effects because OTT is closely related to many factors such as tumor response, dose, fractionation, and treatment-related side effects.

The major limitation of this study is its retrospective nature, with 23 patients receiving EBRT in other institutions before the MR-IGABT in our center. This results in a lack of unity in the EBRT dosimetric data and treatment characteristics. In addition, a longer follow-up may be required to obtain more meaningful results.

## Conclusions

This retrospective study including 97 consecutive Chinese patients with LACC has shown that MR-IGABT was effective and safe. It showed favorable LC, OS, toxicity, and morbidity. Moreover, initial HR-CTV, HR-CTV D_90_, and age were significant prognostic factors. Future investigations and systemic treatment need to be emphasized.

## Data Availability Statement

The raw data supporting the conclusions of this article will be made available by the authors, without undue reservation.

## Ethics Statement

The studies involving human participants were reviewed and approved by the ethics committee of China-Japan Union Hospital of Jilin University. The patients/participants provided their written informed consent to participate in this study.

## Author Contributions

YC: manuscript writing, data collection, and data analysis. YP: protocol development. NZ: manuscript writing. DH: data management. XG: data analysis. ZM: data analysis. GC: protocol development and manuscript editing. All authors listed have made a substantial, direct, and intellectual contribution to the work and approved it for publication.

## Funding

This work was partially supported by grants from the National Natural Science Foundation of China (grant numbers 82073331, 81201737, 31600679, 81703034, 82003208), Project of Science and Technology Department of Jilin Province (grant number 20190303151SF, 20210401138YY), and Horizontal Project of Jilin University (grant numbers 2019YX435, 2019155).

## Conflict of Interest

The authors declare that the research was conducted in the absence of any commercial or financial relationships that could be construed as a potential conflict of interest.

## Publisher’s Note

All claims expressed in this article are solely those of the authors and do not necessarily represent those of their affiliated organizations, or those of the publisher, the editors and the reviewers. Any product that may be evaluated in this article, or claim that may be made by its manufacturer, is not guaranteed or endorsed by the publisher.
